# The Advantage of Automatic Peer-Reviewing of ^13^C-NMR Reference Data Using the CSEARCH-Protocol †

**DOI:** 10.3390/molecules26113413

**Published:** 2021-06-04

**Authors:** Wolfgang Robien

**Affiliations:** Department of Organic Chemistry, University of Vienna, Währingerstrasse 38, A-1090 Vienna, Austria; Wolfgang.Robien@univie.ac.at

**Keywords:** NMR, ^13^C-NMR, computer-assisted peer-reviewing, spectrum prediction, structure generation, database

## Abstract

A systematic investigation of the experimental ^13^C-NMR spectra published in *Molecules* during the period of 1996 to 2015 with respect to their quality using CSEARCH-technology is described. It is shown that the systematic application of the CSEARCH-Robot-Referee during the peer-reviewing process prohibits at least the most trivial assignment errors and wrong structure proposals. In many cases, the correction of the assignments/chemical shift values is possible by manual inspection of the published tables; in certain cases, reprocessing of the original experimental data might help to clarify the situation, showing the urgent need for a public domain repository. A comparison of the significant key numbers derived for *Molecules* against those of other important journals in the field of natural product chemistry shows a quite similar level of quality for all publishers responsible for the six journals under investigation. From the results of this study, general rules for data handling, data storage, and manuscript preparation can be derived, helping to increase the quality of published NMR-data and making these data available as validated reference material.

## 1. Introduction

NMR-spectroscopy is an important technique providing a massive amount of information during the structure elucidation process at the level of the constitution, configuration, and conformation of an unknown compound. The tremendous development of pulse techniques, high-field NMR-equipment, and automatic sample changers during the last three decades has dramatically shifted the earlier bottleneck of the amount of time necessary for acquiring the experimental data to a new bottleneck of spectrum interpretation. This has led to the effect of quite frequent misinterpretations of experimental data in terms of the wrong structure proposals. A systematic analysis of structure revisions was published by Nicolaou [1], pointing out the importance of structure proofs by organic synthesis. Pauli and coauthors [2] demonstrated the necessity of a searchable public domain repository holding the raw spectral data used during the structure elucidation process. A comprehensive review [3] of computer-assisted peer reviewing and subsequent fully automatic structure revisions verified the tremendous effect of automatic quality control for spectral data. 

The flood of experimental NMR data has stimulated the development of computer-software, helping spectroscopists with data interpretation. Carbon-NMR spectroscopy is well-suited for this purpose because of its large range of chemical shift values and its simplicity based on usually missing coupling patterns. A powerful computer-assisted technology for spectrum prediction, named HOSE-code, was introduced in 1978 by Bremser [4]. Later on, neural networks [5] were used; this technology was reprogrammed recently in a similar way and is now called machine learning [6] using specialized hardware architecture. The HOSE-code in its basic version represents only the constitutional properties of the molecule (except for the easy case of cis-/trans-isomerism) and was later on expanded to also handle stereochemical features [7,8]. Meanwhile, stereochemistry can also be used for spectrum prediction in the open-access system nmrshiftdb2 [9]. Since the late 1970’s, many databases collecting the reference material published in the public domain chemical literature were built (ACD/NMR-Predictor [10], CAST/CNMR [11], CSEARCH [12], KnowItAll [13], NMRPREDICT [14], NMRSHIFTDB [15], and Spectrabase [16]). Spectrum prediction engines were also implemented into drawing programs for chemical structures [17], as well as into programs for processing measured NMR-data [18,19].

NMR-spectroscopy is an atom-centered type of spectroscopy, allowing for the assignment of a measured chemical shift value to a specific atom. Understanding this correlation is essential because the value of the chemical shift is influenced by the structural environment of the atom under investigation. This relationship between a structural property and the value of the chemical shift is the basis for all database-oriented prediction tools. Independent from the mathematical model behind the prediction, we need to start from the correct data to describe this correlation. From this simple strategy behind spectrum prediction, it can be immediately derived that publishing simply the list of peaks is not sufficient, because the central information—the 1:1 relationship between structural environment and the chemical shift value—is not furthermore available then. Such an unassigned peak list only represents a fingerprint for a given structure sufficient for spectral similarity searches, but is completely useless for spectrum prediction.

Despite the tremendous development in the field of pulse-techniques available in NMR-spectroscopy, many structure elucidation problems are solved through a comparison of the new measurements with already published reference material taken from the public domain chemical literature. The main problem with the existing reference material is that there is usually no information available about the quality of these data [20]. During the last two decades, many journals have published so-called “Supplementary Material” for their articles—mainly as PDFs—showing, in principle, only pictures of the spectra, efficiently prohibiting the reprocessing of the underlying experimental data. With respect to NMR-measurements, there is an ongoing initiative [21,22] to agree on a common vendor-independent format for the raw data in order to add them to the supplementary material of the publication. This strategy allows for starting a necessary reprocessing and reinterpretation from the original datasets in case of doubt. A well-defined exchange format is furthermore a prerequisite to create community-driven databases [23,24].

It is interesting to observe that many errors in the literature are introduced by misinterpretation of the spectra obtained using state-of-the-art 2D-techniques. A leading role with respect to this effect, seems to be HMBC-type spectra showing frequent misinterpretations of ^2^J (or ^4^J)–couplings, as ^3^J-couplings necessarily leading to incorrect structure proposals [25]. This kind of incorrect interpretation of 2D information is frequently preferred against the application of simple spectrum prediction, efficiently showing the inconsistency between the structure proposal and spectral data.

## 2. Results and Discussion

### 2.1. The CSEARCH-Robot-Referee—General Overview

The automatic structure verification based on ^13^C-NMR chemical shift data is the central procedure within the “CSEARCH-Robot-Referee” [3,26,27,28]. The knowledge base behind consists of some 340,000 curated ^13^C-NMR spectra taken from the public domain literature. A database holding 520 million predicted spectra was additionally used to allow for efficient structure dereplication. The workflow applied here consisted of the following steps:

- Formal check of the structure (valency, charge, and stereocenter);
- Create a table to link the structure to other databases (e.g., PUBCHEM [29,30], Chemspider [31], and eMolecules [32]);
- Check formal correctness of the supplied peak list (symmetry and exchangeable assigned signals);
- Perform spectrum prediction by HOSE-code [4] and NN [5];
- Perform statistical analysis based on underlying data used during the spectrum prediction process to allow for the evaluation of the quality of the result;
- Assign signals if unassigned signals are given;
- Calculate and visualize the coincidence between the structure proposal and the given experimental data;
- Perform a search for identical structures contained in the underlying knowledge base;
- Perform a search for an identical spectral pattern associated with different structures;
- Detect positions with a large deviation between the experimental and predicted values;
- Start structure generator, which modifies the topology of the given structure exclusively at the positions with a large deviation;
- Perform dereplication based on the given peak list using the database of 520 million predicted spectra.

This workflow was applied to 10,039 spectra taken from *Molecules* between 1996 and 2015. The publications having assigned ^13^C-NMR data within this range of years were extracted and these data sets were used to build the CSEARCH database. The reason for selecting this period of time was simply that we were not able to extract more than approximately 25 K spectra per year from the literature, and we had to switch to other journals in order to cover as many journals as possible, with the goal of achieving a high structural diversity. When comparing *Molecules* with other prominent journals in the field of natural product chemistry, like *Chemical and Pharmaceutical Bulletin*, *Fitoterapia*, *Journal of Natural Products*, *Phytochemistry*, and *Planta Medica,* a similar number of compounds were found that seemed to be in error. It should be mentioned that the manually performed extraction of the data from the journal was already quite selective, because datasets that seemed to be in error at a first glance were completely ignored, leading to an improved impression for the respective journal. This effect influences all journals in a quite similar and positive way according to our experience. The ^13^C-NMR data published in these six above-mentioned journals (Table 1) came from compounds of a similar size with an average molecular weight between 431 and 520 amu; the average deviation between the experimental and predicted chemical shift values was also very similar (1.61–2.07 ppm). The number of compounds with at least one signal more than 20 ppm away from the predicted chemical shift value was between 1.34 and 3.40% of the entries available in the CSEARCH-collection. A nearly identical finding was given when the prediction was restricted by a partial structure search; the examples selected here contained either a chromone-fragment or a steroid skeleton. In both cases, the average deviation was again in a very narrow range, starting at 1.40 ppm and 1.27 ppm, respectively, with a maximum at 1.74 and 1.60 ppm, respectively. From the data compiled (Table 1), it can be concluded that all six journals showed a similar level of quality with respect to the ^13^C-NMR data contained therein.

The following examples were taken from articles published in *Molecules* between 2010 and 2015, and were exactly reproduced for input into the CSEARCH-Robot-Referee—any later correction or erratum was ignored, because the intention of this summary is only to show what can be avoided by fully automatic peer-reviewing at the time of uploading a manuscript to the editorial office. The examples are grouped according to the kind of error and only typical cases are shown, far away from being a complete analysis of all published ^13^C-NMR data in *Molecules*. Furthermore, it should be mentioned that this computer-assisted peer-reviewing could be regarded as an effective tool to support the usual peer-reviewing process with respect to quality and time-consumption, going into the details of the structure proofs based on ^13^C-NMR spectroscopy. Throughout the figures, the following coloring scheme is applied in order to increase readability. The first row shows the chemical structure, and in the second row, the structure together with the experimental chemical shift values, as published, are shown, where green highlighting means that signals were assigned by authors and yellow highlighting points to exchangeable signal assignment. The third row shows the differences between the experimental and predicted chemical shift values; deviations between 5 and 10 ppm are highlighted in yellow. Smaller deviations are given in green, whereas larger deviations are shown with red.

### 2.2. Examples of Wrong/Useless ^13^C-NMR Data

#### 2.2.1. Using the Same Data Twice 

**Figure 1:**
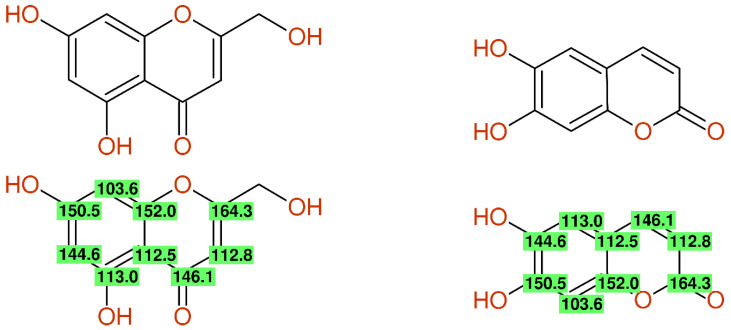

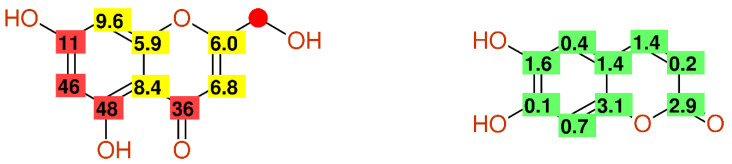
The structures of compound **5** (**left**) and compound **4** (**right**) from [33] together with the published ^13^C-NMR data (green, signal assigned by author, and yellow, signal exchangeable assigned) and the differences between experimental and predicted values (cred > 10 ppm, yellow < 10 ppm and >5 ppm, green < 5 ppm).

In [33], compounds **4** (esculetin; 6,7-dihydroxycoumarin) and **5** (5,7-dihydroxy-2-hydroxymethylchromone) showed different ^1^H-NMR data, but identical ^13^C-NMR chemical shift values were published, as shown in Figure 1. The given values fit to esculetin, but were incomplete and wrong for the proposed chromone-derivative. The structure elucidation was done by comparison with the literature data; therefore it should be mentioned that obviously correct ^13^C-NMR data for compound **5** were already published in [34].

#### 2.2.2. Wrong Values—Strange Substituent Effects

**Figure 2:**
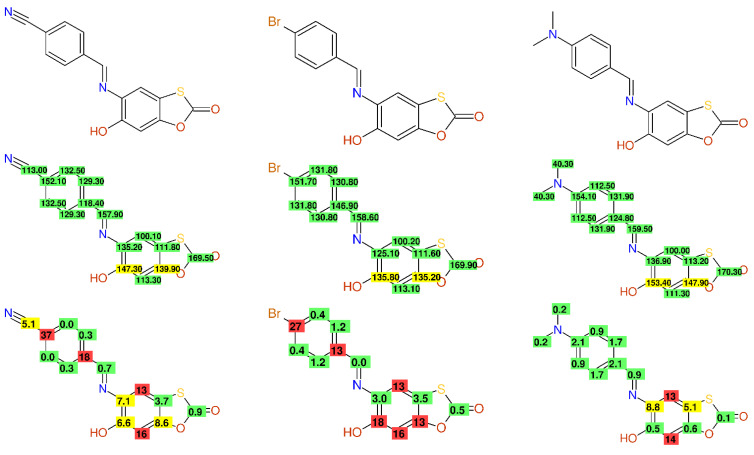
The structures of compound **4a** (**left**), compound **4d** (**middle**), and compound **4i** (**right**) from [35], together with the published ^13^C-NMR data and the differences between the experimental and predicted values.

Bromine-substitution is known to induce a high-field shift of approximately 6 ppm in benzene-derivatives, a more pronounced effect can be observed for a nitrile-moiety (16 ppm); for this reason, the given chemical shift data for the carbon 4’ in compounds **4a** (152.1 ppm) and **4d** (151.7 ppm) published in [35] are far away from the expected values, as can be seen in Figure 2. It is interesting to note that the carbons 6 and 7a in the benzo[d][1,3]-oxathiol-2-one fragment are exchangeable, assigned with values ranging from 135.2/135.8 ppm (compound **4d**) to 153.4/147.9 (compound **4i**); this difference in chemical shift values was obviously attributed, according to the research, to the exchange of a bromo-substituent with a dimethylamino-moiety seven, respectively nine bonds away! The compounds described in this publication contain quite interesting structural features, but the carbon NMR-data are unusable as a high-quality reference material.

**Figure 3:**
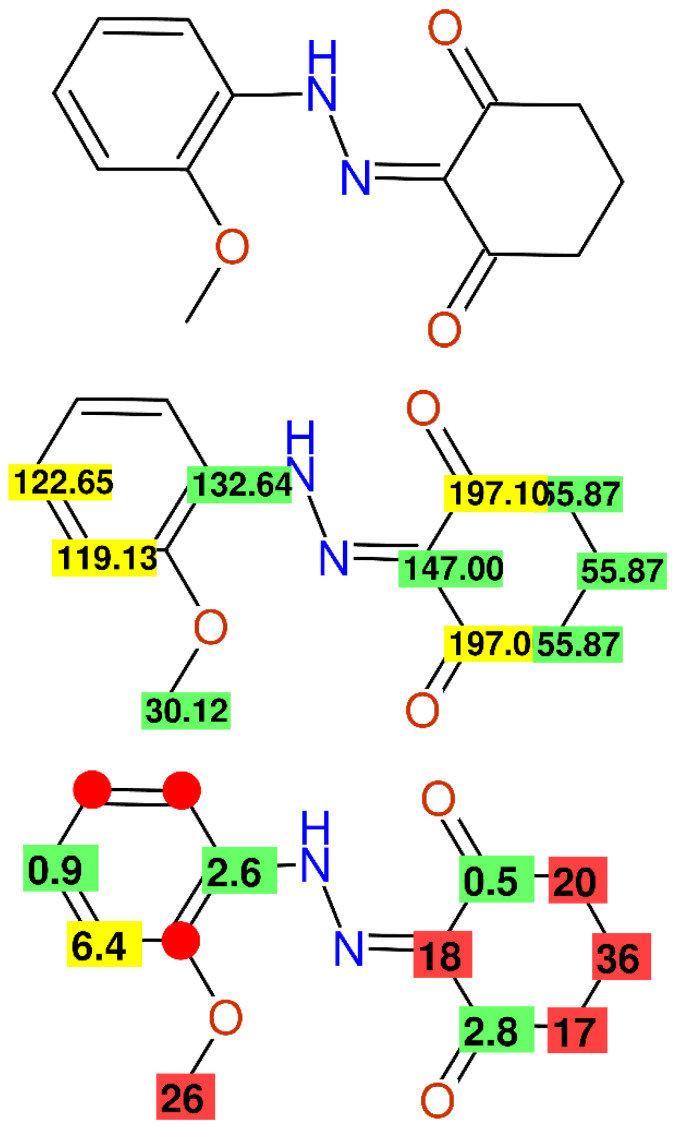
The ^13^C-NMR data of compound **L1** as published in [36], together with the differences between the experimental and predicted values.

In Figure 3, the published NMR-data of ligand **L1** from [36] are summarized, showing a methoxy-group at 30.12 ppm, which is known to resonate within an extremely narrow range of around 56 ppm when one ortho-position is unsubstituted; additionally the chemical shift values of the three CH_2_-groups are far away from the predicted values, and the spectroscopic characterization of the aromatic ring system is incomplete.

**Figure 4:**
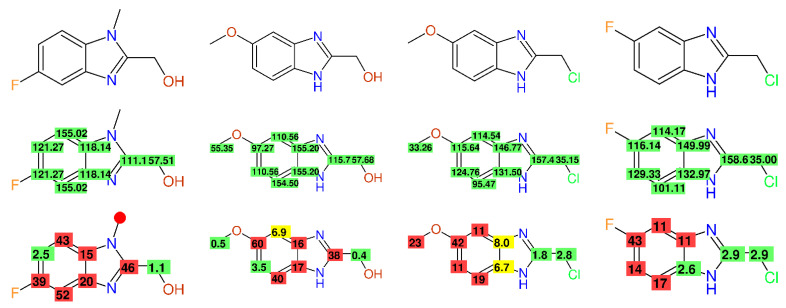
^13^C-NMR data of compounds **51**, **26**, **34**, and **37** (columns from **left** to **right**) as published in [37], together with the published chemical shift values and the differences between the experimental and predicted chemical shift values.

In [37], a series of benzimidazole derivatives was published and the relevant data were compiled in the Supplementary Information. A few compounds contained fluorine, but no C-F couplings in the ^13^C-NMR data were given. In compound **26**, which has two different names (5-Methyl- according to the header, and 5-Methoxy according to the experimental procedure), the aromatic -OCH_3_-group resonated at 55.35 ppm, whereas in compound **34**, a chemical shift value of 33.26 ppm was given. The signal assignment was completely inconsistent within the presented series of compounds—some carbons with a quite small influence to be expected on their chemical shift value covered a range of more than 40 ppm (e.g., C-2). Many compounds showed differences in the range of 40 to 60 ppm between the experimental and predicted values, as given in Figure 4. The overall impression coming from this research was that they were interesting compounds, with ^13^C-NMR data beyond repair.

#### 2.2.3. Typos and Transmission Errors

**Figure 5:**
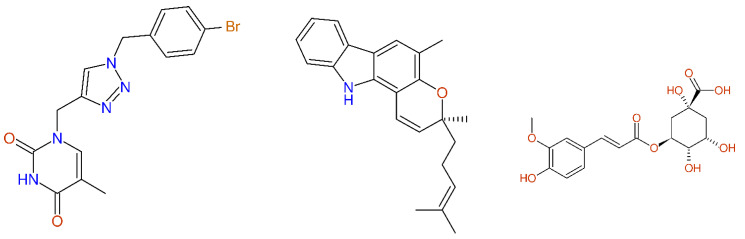

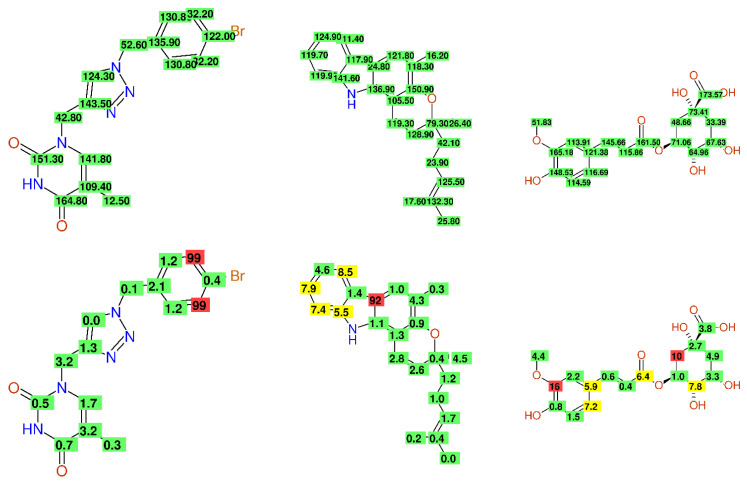
^13^C-NMR data of compound **13** from [40] (**left**), compound **3** (mahanimbine) from [38] (**middle**), and compound **5** from [39] (**right**), together with the differences between the experimental and predicted chemical shift values. Differences larger than 99 ppm are always shown as 99 ppm.

The examples compiled in Figure 5 showed some significant deviations between the predicted and experimental ^13^C-NMR chemical shift values, which could be attributed to typos and transmission errors, because the other shift values were mainly within their expectation ranges. In the case of compound **3** (mahanimbine) from [38] and compound **5** from [39], additional assignment errors could be present, as can be seen in Figure 5 (middle and right column). Compound **5** from [39] shows a quite unusual chemical shift value for an aromatic -O-CH_3_ group (51.83 ppm) making reinspection of the underlying experimental data necessary in order to verify the structural proposal. In the case of compound **13** from [40], a value of 32.2 ppm is given there in Table 3, whereas the experimental part shows a value of 132.2 ppm, as to be expected.

#### 2.2.4. Trivial Assignment Errors

**Figure 6:**
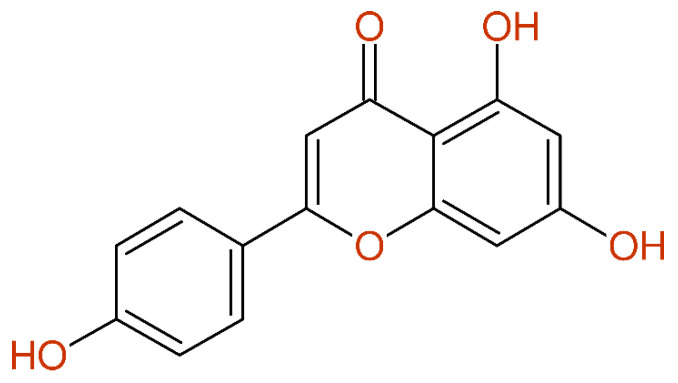

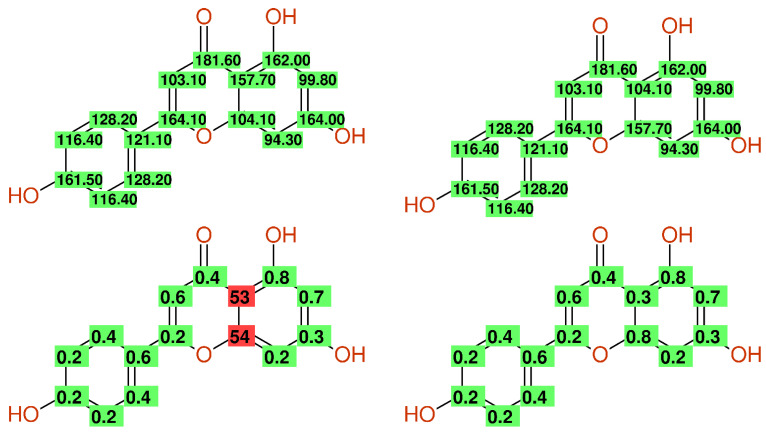
^13^C-NMR data for compound **4** in [41] as published (**left**) and after correction (**right**), together with the differences between the experimental and predicted chemical shift values.

The data of compound **4** (apigenin) from [41] show a typical error based on interchanging the numbering of the structure and the sequence of chemical shift values according to the structure drawing. After this easy correction, an excellent agreement between the predicted and experimental chemical shift values could be achieved, as summarized in Figure 6. The identical problem could be observed with compound **D1** from [42] and compound **12** from [43]. 

Other trivial assignment errors could be found with compound **3** published in [44]; compound **3** from [45]; compound **3** from [46]; compound **2** in [47], despite excellent Supplementary Information; compound **1** from [48]; and compound **3** from [49].

**Figure 7:**
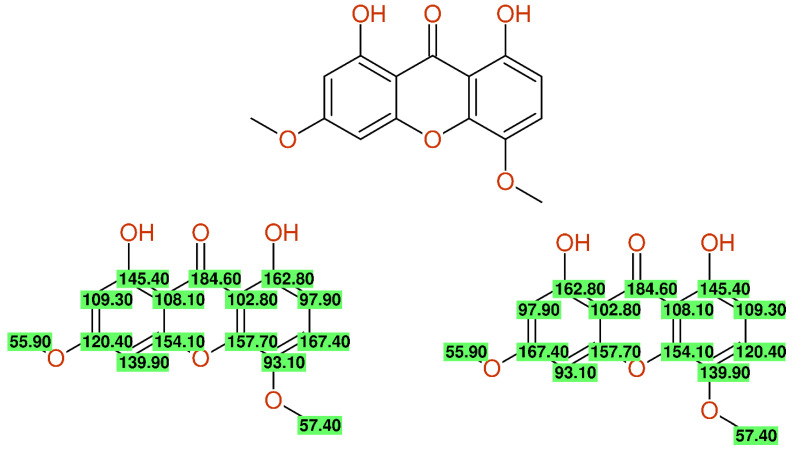

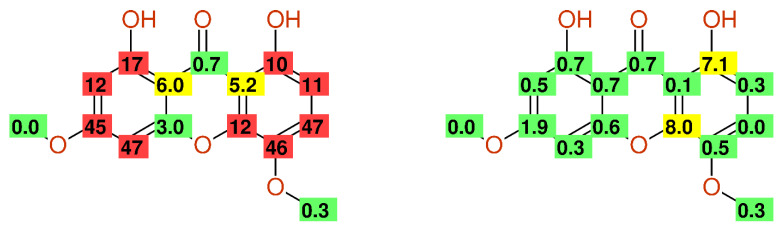
^13^C-NMR data of compound **1** from [50] as published (**left**) and after correction (**right**), together with the differences between the experimental and predicted chemical shift values. The deviations remaining after the first step of correction (7.1/8.0 ppm) point out an additional assignment problem.

A more sophisticated error occurred when the numbering schemes of two complete ring systems were intermixed [50], leading to massive deviations between the experimental and predicted chemical shift values at nearly all positions; in this particular case, another possible assignment error (carbon 1 versus 4a) increased the complexity of the correction, as can be seen in Figure 7.

#### 2.2.5. Alkyl-Chains

**Figure 8:**
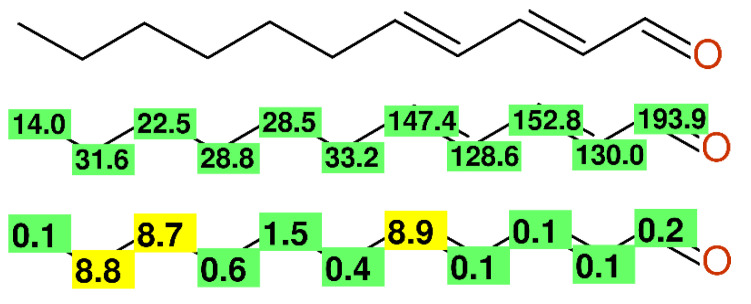
Misassigned alkyl-chain of compound **11** from [51]. Experimental chemical shift values (**middle**) and differences between experimental and predicted chemical shift values (**bottom**).

It is known from basic textbooks on NMR-spectroscopy that an unbranched alkyl chain is characterized by a quartet around 14 ppm, 2 triplets around 22 and 32 ppm, and all of the other more centered CH_2_-groups resonate around 29 ppm. In the chemical literature, there are frequently examples with a different sequence of signals occurring, either caused by typos or wrong assignments. Compound **11** (Figure 8) and compound **12** from [51] showed this misassignment, whereas other compounds (e.g., compounds **4**, **5**, and **9**) from the same paper were correctly assigned.

**Figure 9:**
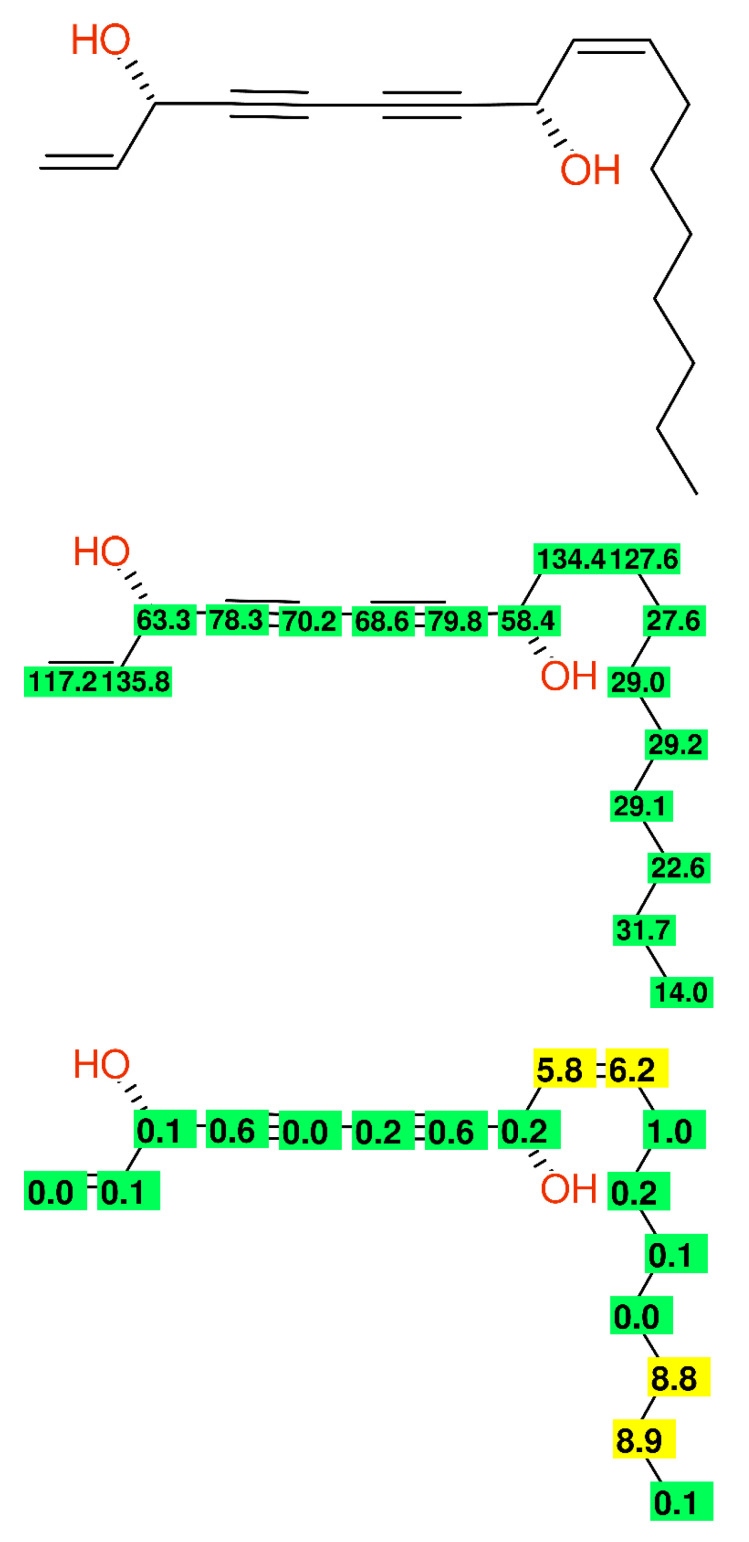
Compound **OH-1** from [52] as another example of a misassigned alkyl chain. Experimental chemical shift values (**middle**) and the differences between the experimental and predicted chemical shift values (**bottom**).

The identical problem with the wrong assignment of an alkyl chain can be observed at compound **OH-1** in [52]; the published data of (3S,8S)-falcarindiol are compiled in Figure 9.

#### 2.2.6. Wrong Structure Drawing

**Figure 10:**
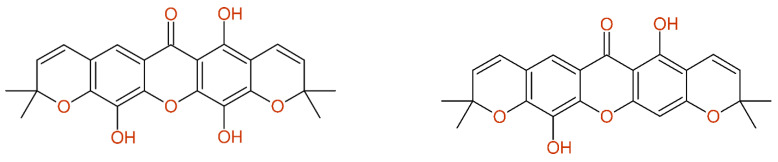

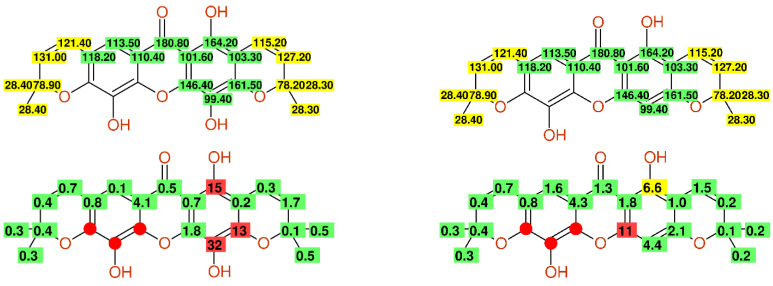
The data of compound **3** from [53] named pyrojacareubine, as published (**left**) and after correction of the structure (**right**), still holding additional assignment errors.

The ^13^C-NMR data of pyrojacareubine (compound **3** in [53]) show severe deviations between the experimental and predicted chemical shift values; the differences were focused into one aromatic ring system, as given in Figure 10. This asymmetric C_23_-compound was characterized by only 20 distinct carbon chemical shift values. The molecular formula was explicitly given as C_23_H_20_O_6_, whereas the structural diagram held seven oxygens. Removing the 4-hydroxy group from the structure resulted in the well-known compound pyranojacareubin, here named “pyrojacareubine”. According to the ^1^H-NMR data, a chemical shift value of 6.27 ppm for H-4 was given, supporting the assumption of a drawing error with respect to an additional hydroxy-group in position 4. Independently of this correction, the problem with three missing signals remained and a minor assignment error was still present. The wrong structure from this publication was used later in an investigation on antimalarial QSAR analysis [54], influencing the results published there. 

#### 2.2.7. Multiple Inconsistencies

**Figure 11:**
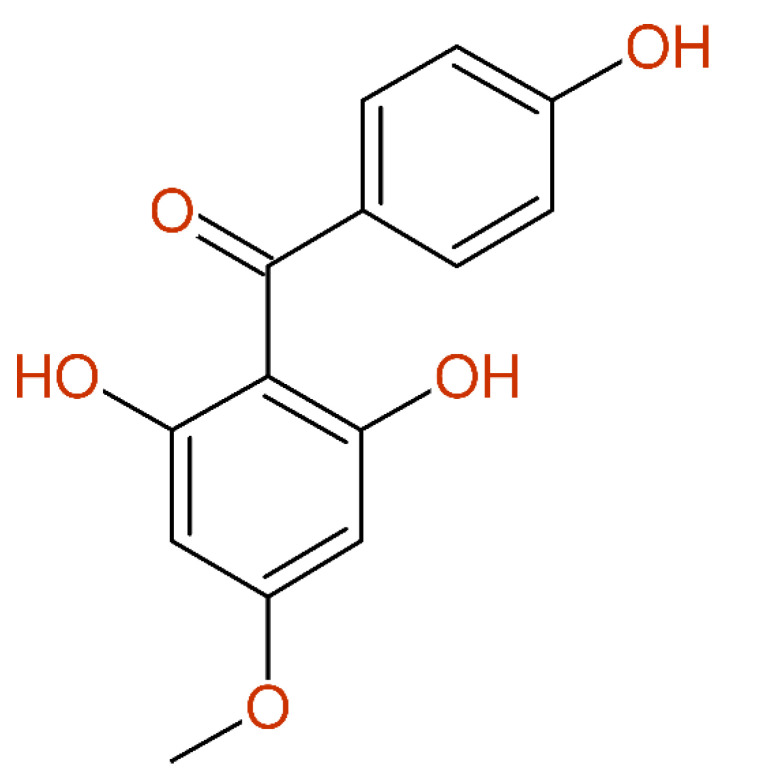

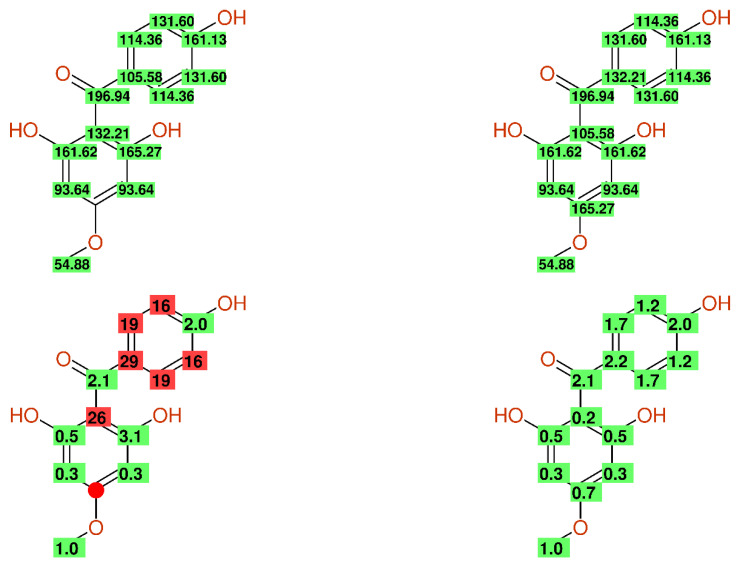
^13^C-NMR data of a benzophenone-derivate (already corrected with respect to the erroneously drawn hydroxy-group in position 2) taken from [55], as published (**left**) and the corrected version (**right**).

In [55], a benzophenone-derivative was published, together with its ^1^H- and ^13^C-NMR data, as a structure proof. The following problems were related with this example. Only the facts freely available in the PDF are summarized here. The numbering scheme was wrong (1 versus 1’), leading to a misassignment of the carbon signals at 132.21 and 105.58 ppm. The compound name was inconsistent with the drawing (drawing had four hydroxy-groups, the name was given as “trihydroxy-“). The signals at 131.60 and 114.36 ppm, as well as the associated ^1^H chemical shift values, were interchanged. According to the ^1^H and ^13^C chemical shift values in positions 3’ and 5’, the symmetry of the aryl-moiety was correctly represented, whereas the carbon chemical shift values in positions 2’ and 6’ reflected non-equivalence and the resonance line of C-4’ was missing. The summary for the presentation of this compound, as shown in Figure 11, is as follows: wrong structure drawing, compound name inconsistent with the structure drawing, wrong numbering, wrong signal assignment, missing line caused by wrong assignment—it is a fact that this paper successfully passed the peer-reviewing procedure.

#### 2.2.8. Fully Automatic Structure Revisions

**Figure 12:**
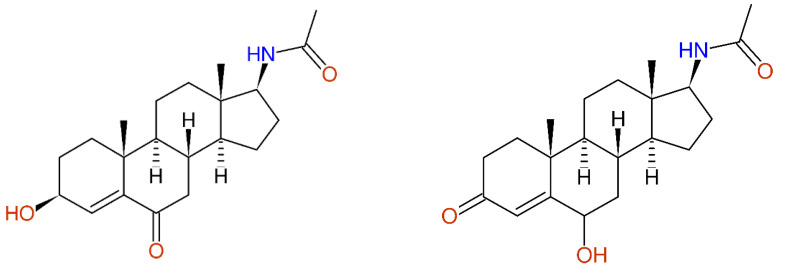

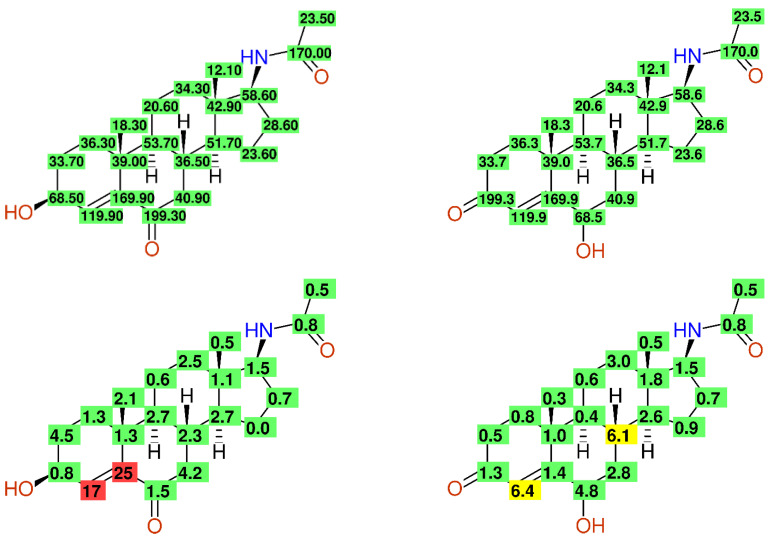
3ß-Hydroxy-17-acetamidoandrost-4-en-6-one as published in [56] for compound **8** (**left**), together with the differences between the experimental and predicted chemical shift values. The automatic structure revision proposes the 6-hydroxy-3-one derivative (**right**) leading to a much better, but not perfect coincidence, with the experimental data.

In [56], the selective reduction of 17-acetamidoandrost-4-en-3,6-dione (compound **6**) using NaBH_4_/NiCl_2_.6H_2_O was published. The product was elucidated as 3ß-hydroxy-17-acetamidoandrost-4-en-6-one (compound **8**), showing the ^13^C-NMR chemical shift values as given in Figure 12. The prediction of the spectral data led to significant deviations for both carbons of the 4,5-double bond, showing some errors during the structure elucidation process. Starting the structure generator with the obviously wrong structure proposal and the given experimental ^13^C-NMR data produced 1591 alternative structures. The given proposal was found at position 358, with an average deviation of 5.20 ppm, whereas the corresponding 6-hydroxy-3-one derivative was ranked at position 2, having an average deviation of only 1.49 ppm. The ^13^C-NMR data of only one stereoisomer of this alternative structure are known [57]—these data slightly differ from those published in [56], showing the necessity of going back to the original measurements to clarify these inconsistencies.

**Figure 13:**
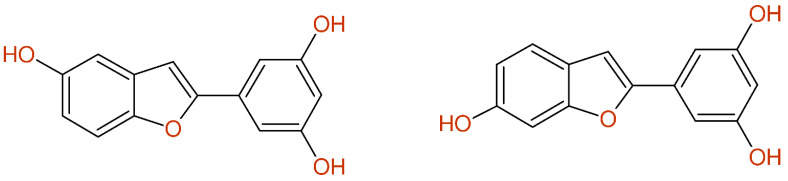

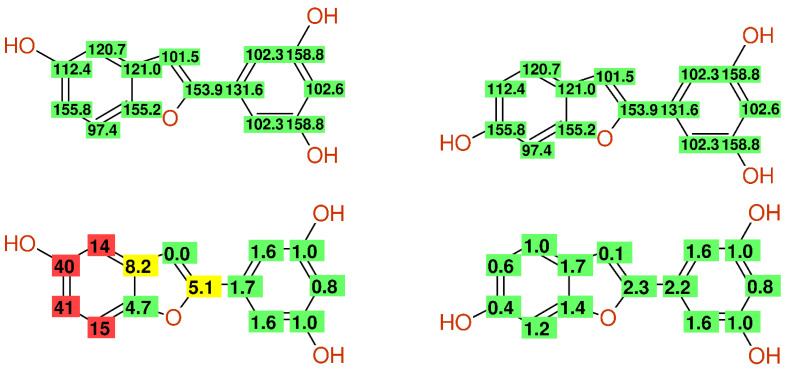
(**left**): Published structure of Moracin M (compound **7** in [41]) together with the given ^13^C-NMR data and the deviations between the experiment and prediction. (**right**): Fully automatic structure revision leading to excellent coincidence between the experimental and predicted chemical shift values.

Another fully automatic structure revision started from the ^13^C-NMR data and the obviously wrong structure proposal (compound **7** in [41]) given for Moracin M, as summarized in Figure 13. The structure generator created 1794 alternative structures, which were sorted by the difference between the experimental and the predicted chemical shift values. The published proposal was ranked at position 66, with an average deviation of 2.46 ppm; however, the correct structure of Moracin M is found at position 1, with an average deviation of only 1.42 ppm. This automatic structure correction was further supported when retrieving the known structure of Moracin M using CAS-Scifinder.

## 3. Conclusions

A similar number of examples of problematic or unusable ^13^C-NMR data can be found in nearly every journal—this is simply an indication of the “publish or perish” mentality concomitant with the preference of quantity instead of quality. From the examples summarized here, the following conclusions can be drawn with the intention of improving the quality of published NMR reference data.

- Structures must be deposited as computer-readable files (e.g., MOLfile)—every structure drawing must be derived thereof in order to avoid drawing errors.
- Every structure must be accompanied by a unique identifier (e.g., INCHIKEYS), avoiding transmission errors and allowing “identical structure search” via text-based search-engines.
- Every NMR-dataset must be validated (e.g., using CSEARCH [26], MNova [18], and ACD [10]).
- Every NMR-dataset that is uploaded (e.g., NMReDATA [21,22]) must be stored in a searchable, public domain, open access, and curated repository—allowing for the automatic detection of “reusing” already known NMR-data in order to verify another structure proposal [58].
- It is highly advisable to combine a database-oriented approach, as described here, with DFT calculations [59,60].
- As many steps as possible in the process of publishing results must be done in a software-supported way. It must be mandatory for authors to provide all of the necessary experimental data (free induction decays for NMR) so that every conclusion can be reproduced, and it must be mandatory for the publishers to allow for the uploading of such data. Furthermore, these data must be made searchable and downloadable for later use.
- Improvements in the actual “peer-reviewing” workflow, including massive computer-supported technologies, every set of spectral data has to be automatically checked during upload, and the associated protocol as described here must be an integral part of the manuscript available to the reviewer(s) and, upon publication, to the readers.

## Figures and Tables

**Table 1:** Comparison of the quality of ^13^C-NMR data published in *Molecules* (MOL) versus *Chemical and Pharmaceutical Bulletin* (CPB), *Fitoterapia* (FT), *Journal of Natural Products* (JNP), *Phytochemistry* (PC), and *Planta Medica* (PM).

Journal	MOL	CPB	FT	JNP	PC	PM
**Entries**	10,039	17,863	1568	34,933	38,379	3621
**Period**	1996–2015	1977–2016	1998–2012	1979–2013	1976–2015	1977–2006
**Average MWT (amu)**	431	520	497	481	483	479
**Δδ_C_ (ppm)**	2.07	1.61	1.85	1.79	1.66	1.72
**Δδ_C_ > 20 ppm**	341/3.40%	239/1.34%	42/2.68%	542/1.55%	533/1.39%	64/1.77%
**Δδ_C_ (ppm) Chromones**	1.59	1.40	1.74	1.68	1.45	1.57
**Δδ_C_ (ppm) Steroids**	1.60	1.27	1.54	1.47	1.37	1.36

## Data Availability

Data available from https://c13nmr.at/journal/molecules/.

## Sample Availability

No samples available.
